# Molecular Profiling of Athletes Performing High-Intensity Exercises in Extreme Environments

**DOI:** 10.3390/sports11020036

**Published:** 2023-02-02

**Authors:** Kristina A. Malsagova, Arthur T. Kopylov, Alexander A. Stepanov, Dmitry V. Enikeev, Natalia V. Potoldykova, Evgenii I. Balakin, Vasiliy I. Pustovoyt, Anna L. Kaysheva

**Affiliations:** 1 Biobanking Group, Branch of Institute of Biomedical Chemistry “Scientific and Education Center”, 109028 Moscow, Russia; 2 Institute for Urology and Reproductive Health, First Moscow State Medical Sechenov University, 119992 Moscow, Russia; 3 State Research Center–Burnasyan Federal Medical Biophysical Center of Federal Medical Biological Agency, 119435 Moscow, Russia

**Keywords:** metabolome, athletes, ELISA, mass spectrometric analysis, IgG, food allergens, amino acids

## Abstract

The aim of this study was to determine the influence of high-intensity training under extreme conditions (T = 40 °C) on the metabolism and immunological reactions of athletes. Male triathletes (*n* = 11) with a high level of sports training performed load testing to failure (17 ± 2.7 min) and maximum oxygen consumption (64.1 ± 6.4 mL/min/kg). Blood plasma samples were collected before and immediately after exercise. Mass spectrometric metabolomic analysis identified 30 metabolites and 6 hormones in the plasma, of which 21 and 4 changed after exercise, respectively. Changes in the intermediate products of tricarboxylic and amino acids were observed (FC > 1.5) after exercise. The obtained data can be associated with the effect of physical activity on metabolism in athletes. Therefore, constant monitoring of the biochemical parameters of athletes can help coaches identify individual shortcomings in a timely manner and track changes, especially as the volume of training increases. In addition, it was revealed that the immunological reaction (manifestation of a hyperactive reaction to food components) is personalized in nature. Therefore, it is important for coaches and sports doctors to analyze and control the eating behavior of athletes to identify food intolerances or food allergies in a timely manner and develop an individual elimination diet.

## 1. Introduction

Studies on the molecular mechanisms that accompany adaptation to physical activity are becoming increasingly popular [1,2].

Changes in the blood metabolic profile in professional athletes are preceded by overtraining, which largely depends on the variant and duration of the training mode [3]. During training, various behavioral, biochemical, hormonal, and immune markers are used to assess the physiological condition of athletes [4]. However, some studies have demonstrated that standard tests cannot accurately detect physiological pre- and postexercise differences between endurance athletes and controls [5].

The aim of metabolomics is to quantify the profiles of endogenous non-molecular components associated with exercise in professional athletes in order to identify biomarkers of performance, fatigue responses, and possibly sport-related disorders [5,6]. Metabolomics allows for the detection of changes in response to various physiological stimuli and helps to identify metabolic traits with potential translational impacts in professional athletes [7]. These changes include the metabolic products associated with the metabolism of glucose, lipids, amino acids, and energy [3,5]. In addition, metabolomic profiling of athletes performing intense exercise revealed changes in plasma lactate [8] and breakdown products of nitrogenous bases, in particular adenine [9], indicating anaerobic metabolism and ATP production, respectively. Howarth et al. examined changes in the level of the Krebs cycle or the TCA cycle (tricarboxylic acid cycle) intermediates [10]. Increases in serum sex steroid hormones have also been reported in endurance athletes in response to high-intensity exercise [11].

To determine the hyper-reactive reaction to food allergens mediated by immune processes, an enzyme-linked immunosorbent assay (ELISA) was performed before and after exercise to determine the concentration of class G immunoglobulins (IgG). ELISA is widely used in allergology and immunology for the diagnosis of antigen–antibody-type immune reactions with high selectivity, reproducibility, and specificity [12,13]. In immunological tests, the IgG or G4 subclass is a marker of a specific humoral immune response, which is specific to the food antigen being tested [14,15]. The choice of this class of antibodies is based on the fact that IgG makes up 70–80% of all blood immunoglobulins and plays a fundamental role in providing long-term humoral immunity. Although IgGs are not classical secretory antibodies, they are present in the intestinal lumen, with a protective function. IgGs have been shown to bind to Fc receptors on intestinal epithelial cells in acidic environments. This transcytosis-mediating receptor mediates protective IgG transport and release on the basal side of enterocytes, where neutral pH causes them to dissociate from the receptor [14,15].

IgG is the main antibody of the secondary immune response to most antigens and manifests itself in the form of type III immunopathological reactions. Moreover, IgG ELISA is characterized by a sensitivity of 92–95%, a specificity of 86–89%, and a reproducibility of 95–97%, as well as the possibility of studying the dynamics of the state of the immune system before and after an elimination diet [16].

Elite-level athletes often perform under extreme environmental conditions, such as high altitude and extremely high or low air temperatures. Performance under such conditions may be accompanied by changes in the molecular profile of athletes, as well as the development of stress characterized by low performance and low efficiency of recovery. Furthermore, the adaptation of athletes to extreme conditions is probably individual and depends on many factors. Many studies have been conducted with the aim of monitoring the state of the bodies of athletes training under normal conditions. However, physiological profiles are mostly compared by competitive level or across disciplines [17,18]. The content of various xenobiotics or other additives in biological samples of athletes in various sports is often studied [19,20]. Studies on changes in the molecular profile of athletes training in extreme conditions are limited.

The aim of this study was to identify the metabolic characteristics associated with endurance loading under extreme conditions. It was hypothesized that evaluating these metrics can provide valuable insights into athletes’ current fitness and training adaptations. A deeper understanding of the quantitative changes in low-molecular-weight (endogenous) participants in biochemical processes (tricarboxylic acid cycle, protein metabolism, ornithine cycle, etc.) during physical activity can allow for the design of comprehensive training programs that prevent potential overexercise disorders and improve general performance by changing the signature of endogenous metabolites.

## 2. Materials and Methods

### 2.1. Study Participants

This study involved 11 male athletes involved in a multisport endurance race consisting of swimming, cycling, and running over various distances (triathlon). The anthropometric characteristics of the participants are listed in Table 1.

The functional characteristics of the participants are presented in Table 2.

Inclusion criteria:

(1) Qualifications of an athlete must be proven during the year before inclusion by participating in competitions during the winter season up to three times and in summer races up to four times.

(2) High-intensity physical activity at aerobic and anaerobic thresholds (80% and 20%, respectively) carried out for 2 h three times a week, with speed–strength training carried out for 2.5 h twice a week.

(3) At the time of the study, the doctor performed a comprehensive assessment of athletes’ health status to issue a conclusion about their health. The conclusion was obtained based on an in-depth medical examination, which included X-ray examination of the chest, ultrasound diagnostics of the abdominal cavity and pelvic organs, diagnostics of the cardiovascular system, echocardiography, electrocardiography, biochemical tests of urine and blood, and examinations by specialized physicians (ophthalmologists, otolaryngologists, surgeons, cardiologists, neurologists, dentists, endocrinologists, and therapists).

(4) Training was absent for 3 days prior to the start of the study.

All participants were informed of the risks and discomforts associated with the investigation and signed a written consent to participate. The study was approved by the Board for Ethical Questions at the A. I. Burnazyan State Research Center of the FMBA of Russia (Protocol No. 40, 18 November 2020).

### 2.2. Stress Testing

Introductory testing of these groups was performed on separate days. Basic load testing “to failure” was carried out at a temperature of 22 °C and a relative humidity of 60%. During testing, the participants were dressed in sports shorts, socks, and shoes. Testing was continued until volitional exhaustion, defined as the point at which participants were unable to maintain treadmill speed and continue with the load.

Load testing was performed on a V-ergo PRO treadmill (Italy) in a climate-controlled room. Throughout the examination of the participants, ECG parameters were continuously recorded using a Cosmed “Quark C 12” wired recorder to measure the electrical activity of the heart. Indicators of pulmonary ventilation and metabolic response were continuously measured using Cosmed “Quark CPET” (Italy) systems for analysis of the gas composition of inhaled and exhaled air.

Physical activity on the V-ergo PRO treadmill was carried out in accordance with the protocol and continued until the moment of “failure” (Table 3). Subsequently, the recovery phase was activated in the gas analysis program, accompanied by a slowdown in the speed of the moving web to a complete stop after 3 min.

To determine the physical performance and functional reserves of the participants, the following indicators were taken into account: total time of work, time of the onset of the aerobic threshold, time of anaerobic threshold, resting heart rate (RHR) before the start of the test, RHR at aerobic threshold, RHR at anaerobic threshold, RHR maximum, RHR recovery after 5 min, oxygen consumption, removal of carbon dioxide (CO_2_.), respiratory coefficient, metabolic units, frequency of respiratory movements, minute volume of breath, tidal volume, oxygen pulse, respiratory equivalent for O_2_, and respiratory equivalent for CO_2_.

### 2.3. Plasma Samples

Blood samples were collected on an empty stomach from the cubital vein following overnight fasting and stored in vacutainers containing 3.8% sodium citrate anticoagulant (IMPROVACUTER, Guangzhou Improve Medical Instruments Co., Ltd., Guangzhou, China). The samples were centrifuged at 3000 rpm for 6 min at room temperature. Each plasma sample (500 μL) was finally collected into two dry Eppendorf-type polypropylene test tubes, frozen, and stored at −80 °C prior to analysis.

### 2.4. Mass Spectrometric Analysis

Data acquisition was performed using a high-resolution quadrupole time-of-flight (Q-TOF) Xevo G2-XS mass spectrometer (Waters, Inc., Ireland, Milford) equipped with a Z-spray ionization source coupled with a UPLC Acquity H Class (Waters, Inc., Ireland, Milford) system. Details of instrumental analysis are available in [21].

### 2.5. ELISA

Immune response to food ingredients was assessed by a solid-phase, non-competitive, indirect enzyme-linked immunosorbent assay (ELISA) using a commercial reagent kit for a semiquantitative enzyme immunoassay of allergen-specific IgG antibodies (LLC NPO Immunoteks, Stavropol, Russia). This kit uses monoclonal anti-IgG antibodies included in the peroxidase conjugate that are capable of detecting antibodies of the immunoglobulin G class in human serum/plasma, which have an affinity for allergens sorbed on the surface of a polystyrene tablet. The analysis was performed according to the recommendations of manufacturer. The kit contained 2 polystyrene 96-well plates with food antigens immobilized on the surface. One kit was designed to determine the content of specific IgG in eight test samples for 22 allergens (Table 4).

ELISA was performed on a Multiscan FC microplate photometer (Thermo Scientific, Waltham, MA, USA), and the results interpreted according to the manufacturer’s instructions (Table 5).

### 2.6. Statistical Analysis

The metabolite concentrations after recovery and exercise were determined for each study participant. If the value was in the range of (0.67, 1.5), the data were excluded from further analysis, that is, the calculation of the mean and standard deviation. Statistical analyses were performed using R Project for Statistical Computing [22]. A semiquantitative assessment of hormone content was performed taking into account the chromatographic peak area.

## 3. Results

### 3.1. Analysis of the Metabolomic Profile of Participants before and after Exercise

Mass spectrometric analysis of blood plasma revealed that 21 and 4 subjects significantly differed in the content of amino acids and hormones, respectively, and the levels after exercise differed by 1.5 times relative to the level “before exercise” (Table 6).

Table 6 shows that after exercise, an increase in the concentration of 8 metabolites and 2 hormones and a decrease in the concentration of 13 metabolites and 2 hormones were detected in the blood plasma of the participants.

### 3.2. Hyper-Reactive Reaction to Food Allergens Mediated by Immune Processes

**Figure 1:**
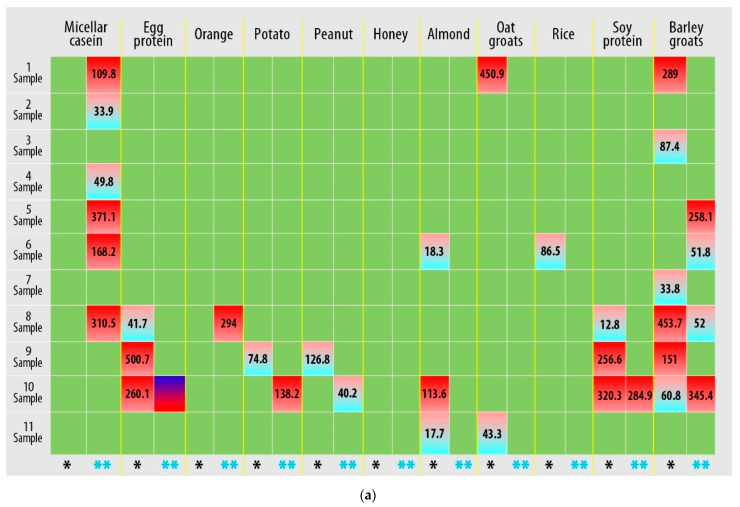

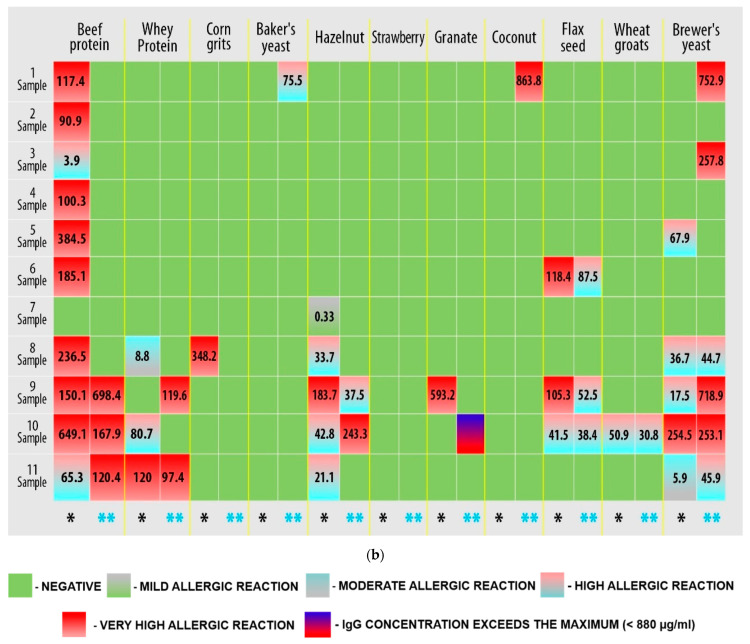
Results of quantitative determination of the content of IgG class antibodies in the blood plasma of athletes (three technical repetitions): (**a**) first plate; (**b**) second plate. Allergens are presented for which the IgG concentration was greater than 0.88 µg/mL (hyper-reactivity to food allergens). Markings: *—immunological reaction before the load; **—immunological reaction after exercise.

Based on the detection of IgG-mediated immunological reactions to 22 food antigens in plasma samples, it was found that all study participants had an allergic reaction to food components (Figure 1, Supplementary S1, Figure S1).

Figure 1 shows that hyper-reactive reactions to food allergens are personalized, and changes in the immunological response must be assessed individually. However, a response to micellar casein (samples 1, 2, 4–6, and 8) and leveling (samples 1–6 and 8) and a decrease (sample 10) in the response to beef protein can be observed after exercise.

**Figure 2:**
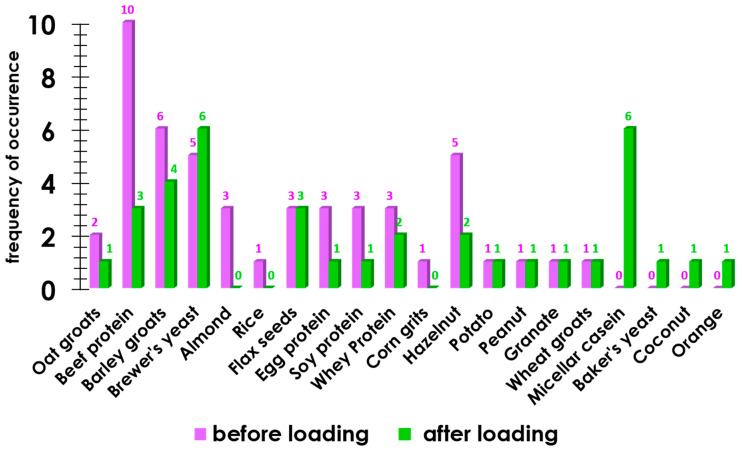
The occurrence of an allergic reaction to food components before exercise and after exercise.

The frequency of hyperactive reactions to food allergens in the study group was also determined (Figure 2).

Figure 2 shows that in 9 out of 11 cases, after the load, the immunological reaction in the group of participants decreased, whereas in 5 cases, the load led to a bodily reaction to food components.

**Figure 3:**
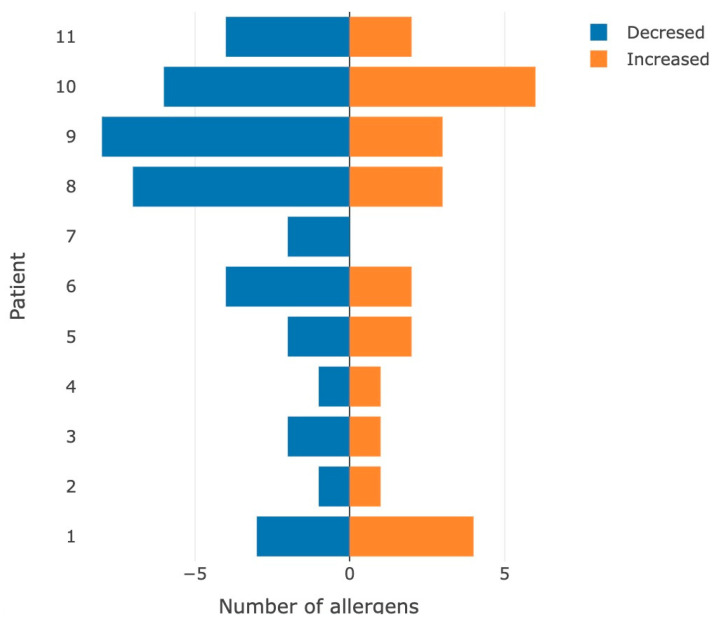
Number of allergens for which the appearance/increase (orange color) or leveling/decrease (blue color) in the immunological hyperactive reaction was detected after performing the stress test.

In general, the load under extreme conditions often led to a decrease in the immunological response (Figure 3).

These results may be associated with a decrease in the concentration of IgG in the blood plasma of participants after exercise due to immunological stress.

## 4. Discussion

**Figure 4:**
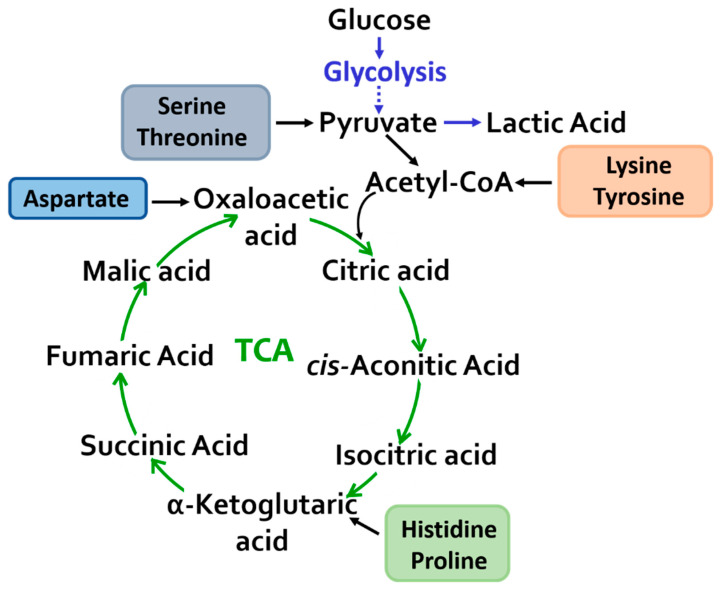
Mitochondrial metabolism: a change in the content of metabolites of the tricarboxylic acid cycle and glycolysis was revealed.

The change in the concentration of intermediates of the TCA cycle is probably associated with the need to meet increased energy requirements during physical exertion (Figure 4) [23].

An increase in the level of amino acids involved in the conversion of pyruvate–serine and threonine and a decrease in the concentration of lysine and tyrosine, which enter an oxidation reaction with the formation of acetyl-CoA, directly feed the TCA cycle. Tyrosine may also be an indicator of exercise-induced adaptation and increased physical activity [24,25,26].

Changes in the content of anaplerotic amino acids (aspartic acid, histidine, and proline) in the blood plasma were recorded and converted into intermediate products of the tricarboxylic acid cycle, thereby replenishing and maintaining the metabolic capabilities of the cycle.

Aspartate, along with other amino acids (leucine, isoleucine, valine, asparagine, and glutamate), is involved in the formation of amino groups and, possibly, ammonia, which are necessary for the synthesis of glutamine and alanine, which are released in excess amounts after absorption and during the consumption of meals containing protein. During physical activity, alterations in profiles of amino acids and endogenous metabolites involved in the Krebs cycle play a key role in the energy metabolism of muscle tissue.

Histidine metabolism, to a greater extent, is directed toward protein metabolism, and catabolism is directed toward glutamate, which can participate in transformations through the formation of α-ketoglutaric acid. The main histidine derivatives present in the human body are 3-methylhistidine, 1-methylhistidine, and ergothioneine. Of these, 3-methylhistidine is formed as a result of post-translational methylation of histidine residues of actin and myosin, which are the major myofibrillar proteins. During protein catabolism (degradation of proteins to amino acids), 3-methylhistidine is released but cannot be reused. Therefore, plasma concentrations of 3-methylhistidine and urinary excretion are sensitive markers of myofibrillar protein degradation [27].

In the present study, a decrease in the level of 3-methylhistidine in the blood plasma of athletes was observed. Information regarding the level of 3-methylhistidine in the plasma is contradictory. Dohm et al. reported that 3-methylhistidine excretion increased in humans after running 12 miles [28]. Other researchers assessed the excretion of 3-methylhistidine after a 100 km run and found no changes compared to the control group at rest [29]. In a study [30] of participants who performed exercises with a maximum load for 3.75 h, a decrease in the level of 3-methylhistidine was found; similar results were obtained in [31]. Considering these variation in results reported by different research groups, we concluded that an increase or decrease in 3-methylhistidine excretion may depend on the type of exercise and the conditions under which it is performed.

Histidine and beta-alanine obtained either from food or by degradation of a pyrimidine base (uracil) in the liver, are the source of carnosine synthesized in support of ATP hydrolysis [32]. Elevated muscle carnosine concentrations are thought to have an ergogenic effect and reduce fatigue during high-intensity physical activity [33]. Glycolytic muscle fibers have a higher level of carnosine than slow twitch (oxidative) fibers due to their primary role in anaerobic exercise and the putative role of carnosine as a pH-buffering smoother [34,35]. In addition, this metabolite acts as an absorber of oxidative stress products in muscles, especially during lipid peroxidation [36]. In the present study, carnosine levels were reduced in athletes after exercise.

There was also a change in the levels of major TCA intermediates such as α-ketoglutaric acid, cis-aconitic acid, citric acid, oxalic acid, and succinic acid.

L-citrulline is an amino acid involved in the urea cycle and may promote the excretion of ammonia and faster recovery of creatine phosphate after exercise. Intracellular accumulation of ammonia promotes glycolysis by inhibiting aerobic pyruvate utilization [37,38] and subsequent lactate formation, which can contribute to fatigue [39,40]. Lactate is the most measurable metabolite, as its level is one of the predictors of the onset of overtraining [41]. Elevated blood lactate levels are a consequence of performing high-intensity exercise and increasing the rate of glycolysis [40]. The concentration of lactate in various body fluids often increases after exercise; however, in the present study, a decrease in plasma lactate levels was observed after high-intensity exercise at high temperatures. Endurance training influences oxidative and gluconeogenic lactate removal [42,43]. Oxidative capacity is enhanced by an increase in mitochondrial density and oxidative enzyme activity. An increase in the proportion of M-type lactate dehydrogenase isoenzymes that promote lactate oxidation is accompanied by adaptations induced by metabolic training [44]. Thus, the use of lactate as a metabolic fuel in the TCA increases with training.

In our study, in which participants performed endurance testing, there was a decrease in creatinine levels. Creatine and creatine phosphate are important sources of energy for athletes during intense exercise. In power athletes, during the load period, the level of creatine increases, and the level of guanidino acetate precursor decreases [41].

We also observed a moderate growth of the blood ascorbic acid level within a few hours after exercise and a subsequent significant reduction (below preworkout levels) of the level a few days after a long training phase. These changes may be associated with increased oxidative stress caused by exercise [45].

Hydroxyproline (Hyp) is a non-essential proteinogenic amino acid that is required to maintain collagen. Therefore, an increase in the Hyp level in blood or urine can be utilized as an indicator of muscle collagen breakdown [46,47]. For example, the authors of [48] observed an increase of up to 69% in urinary Hyp concentration two days after eccentric muscle contraction, indicating the destruction of collagenous connective tissues. In addition, Tofas et al. [47] found that plasma Hyp concentration increased after 24–72 h and peaked (80% increase) 48 h after 200 plyometric jumps, indicating a strong effect on connective tissues. In our study, we observed a decrease in the level of Hyp in the blood plasma of sportsmen immediately after exercise, which is presumably associated with a reduced concentration of proline, as the immediate precursor of hydroxyproline is proline.

Taurine is the product of metabolism of sulfur-containing amino acids (methionine, cysteine, homocysteine, and cystine). It is found in muscles in relatively high concentrations. Taurine plays a role in the maintaining normal contractile function. Evidence suggests that this function of taurine is mediated by changes in the activity of key Ca^2+^ transporters and modulation of the Ca^2+^ sensitivity of myofibrils [49].

The source of elevated plasma taurine after exercise remains debatable, as it may reflect muscle tissue damage, muscle overtraining, or adaptation to changes in blood osmolarity. High concentrations of taurine are found in different muscle types. This illustrates that specific carriers and channels do exist in muscle membranes to support the import and export of taurine. Skeletal muscle remains the most likely candidate to explain the increase in plasma taurine concentration following endurance exercise.

In [50], plasma taurine levels peaked immediately after exercise was stopped. This was confirmed in the present study (Table 6).

Exercise affects the level of sex steroids, including testosterone, in both non-athletes and athletes [11,51]. In this study, an increased level of steroid hormones was observed in high-endurance athletes. Elevated steroids included 11-deoxycortisol and estriol (E3).

During maintained high-intensity physical activity, an increased level of circulating blood metabolites is observed, although the effect is ultimately short-term [52]. Sex steroid hormones play a critical role in biochemical processes, including metabolism of glucose, synthesis of muscle protein, and regulation of redox homeostasis [11]. Steroids stimulate muscle mass growth, energy production, and excitability of the nervous system, increasing the endurance of the body. Athletes who participated in this study successfully passed doping control tests, so changes in steroid levels may reflect an increased secretion of endogenous anabolic steroids, adaptation to physical activity, and an increase in food intake.

Proper preworkout nutrition is essential for the effectiveness of both workout and performance. Nutrition provides the so-called “energy core” of the workout. Post-workout nutrition is important to improve the recovery that begins immediately after a workout. Proper nutrition after training ensures that the body is doing all that is required for the recovery and adaptation to a previous load. In this study, we analyzed the IgG content in the blood plasma of athletes before and after exercise under extreme conditions (running to failure at T = 40 °C). We found that the reaction of athletes’ bodies to food components is personalized; however, in general, a slight decrease in the immunological response can be observed after exercise.

According to some researchers, serum immunoglobulin levels do not change significantly after exercise [53,54]. However, other authors have made unique observations in the study of the level of immunoglobulins; changes in total serum immunoglobulin after a run of less than 40 km are statistically insignificant, although the concentration of IgG decreases 1.5 h after exercise [55]. Pershin and coauthors described a significant decrease and, in some cases, the almost complete disappearance of immunoglobulins from the blood or saliva at certain points, with very sharp fluctuations in concentration, which were explained by the authors by the binding of immunoglobulin molecules by blood cells and possibly by other cells through Fc receptor intestinal epithelial cells with changes in hormonal and temperature balance and acid–base balance [56]. In addition, Nieman and Nehlsen-Cannarella showed that intense ultramarathon running can lead to a more pronounced and prolonged decrease in serum immunoglobulin levels than exercise of shorter duration. Immunoglobulin IgA and IgG normally found in airway and alveolar secretions may non-specifically diffuse from the serum during recovery from prolonged endurance exercise or in response to microbial agents and antigens introduced into the respiratory tract during exercise [55].

We assume that the obtained results may be due to a shift in the compensatory reactions of the immune system to prioritize defense and adaptation mechanisms. However, because some athletes exhibit a hyperactive reaction to food allergens even before exercise, it is recommended that coaches and sports doctors conduct such an analysis to develop elimination diets for athletes. For a more reliable assessment, it is necessary to adjust the nutrition of athletes based on the data obtained after exercise and subsequent monitoring of immunological reactions under the same conditions after some time (2–3 months). This approach allows for the evaluation of the effectiveness of elimination diets and enables additional adjustments to be made to the athlete’s diet.

## 5. Conclusions

The metabolic blood profile in response to exercise reveals unique traits associated with the type and duration of exercise [3,7]. However, sport metabolomics require further development. The study of metabolic patterns of athletes enables determination of the molecular mechanisms underlying changes in the body state. Alternatively, they could be used as potential biomarkers for sports. In this study, metabolomic analysis was used to determine the metabolite composition of the blood plasma of elite athletes who participated in national or international sports competitions after successfully passing antidoping tests. Despite the paucity of information about the lifestyle of the studied participants and possible factors affecting their metabolic patterns, new data revealed significant differences in the level of metabolites before and after stress testing under extreme conditions. This study demonstrates the relevance of immunological testing for food allergens, which is important because the combination of dyes, preservatives, and hardeners in the composition of products used by humans, including dietary supplements, can lead to an overload of the immune system and, as a result, to the development of numerous chronic diseases. With a single consumption of such products, the formation of an excess of immune complexes does not lead to noticeable disturbances in the functioning of the body. However, the constant presence of such products in the diet leads to a significant increase in the concentration of immune complexes in the blood, an ultrahigh load on the immune system, and the potential occurrence of many individual chronic diseases.

## 6. Limitations

The main limitation of this study is the lack of data on the diets of the athletes, which are required to substantiate the relationship between hyperactive reactions to foods and physical activity in more detail.

## Figures and Tables

**Table 1:** Anthropometric characteristics of study participants.

Sample No. #	Age (Years)	Weight (kg)	Height (cm)	BMI (kg/m^2^)
1	28	82.5	179	25.7
2	28	67	176	21.6
3	28	76.4	180	23.6
4	29	70.8	181	21.4
5	26	72.4	176	23.4
6	28	76.3	180	23.5
7	28	69.0	177	22.0
8	29	80.0	178	22.9
9	32	63.6	171	21.8
10	27	72.9	171	24.9
11	32	82.2	179	25.6

**Table 2:** Functional indicators of study participants.

Indicator	Unit	Mean (SD)
VO_2_ max	mL/min/kg	64.1 ± 6.4
RQ	relative units	1.14 ± 0.1
METs	relative units	18 ± 1.9
Resting heart rate	bpm	82 ± 6.5
Aerobic threshold		
VO_2_	mL/min	47.2 ± 9
VE	L/min	90 ± 17.8
Heart rate	bpm	154 ± 12
Anaerobic threshold		
VO_2_	mL/min	58 ± 9.2
VE	L/min	138 ± 21.5
Heart rate	bpm	176 ± 7.6
Maximal oxygen consumption		
VE	L/min	174 ± 11.4
Heart rate	bpm	186 ± 5.5

Abbreviations: bpm—beats per minute; METs—metabolic equivalents; RQ—respiratory quotient; VO_2_—oxygen consumption.

**Table 3:** Treadmill load protocol *.

Stage	Speed (km/h)	Time (min)
1	5.00	0.00–1.40
2	6.00	1.40–3.20
3	7.00	3.20–5.00
4	8.00	5.00–6.40
5	8.00	6.40–8.20
6	10.00	8.20–9.00
7	11.00	9.00–10.40
8	12.00	10.40–12.20
9	13.00	12.20–14.00
10	14.00	14.00–15.40
11	15.00	15.40–17.20
12	16.00	17.20–19.00
13	17.00	19.00–20.40
14	18.00	20.40–22.00
15	19.00	22.00–23.40
16	20.00	23.40–25.00
Recovery	4.00	2.00
	2.7	3.00

* Treadmill load is a modification of an electrocardiographic examination in which an electrocardiogram is recorded during intense physical activity.

**Table 4:** Food allergens in the reagent kit for a semiquantitative enzyme immunoassay for allergen-specific IgG antibodies.

No.	Allergen	No.	Allergen
1.	Milk protein concentrate (micellar casein)	12.	Beef protein hydrolysate (beef protein)
2.	Dry egg white (egg protein)	13.	Whey protein concentrate (whey protein)
3.	Orange	14.	Corn grits
4.	Potato	15.	Baker’s yeast
5.	Peanut	16.	Hazelnut
6.	Honey	17.	Strawberry
7.	Almond	18.	Pomegranate
8.	Oat groats	19.	Coconut
9.	A mixture of brown and wild rice	20.	Flax seed
10.	Soy protein isolate (soy protein)	21.	Wheat groats
11.	Barley groats	22.	Brewer’s yeast

**Table 5:** Interpretation of ELISA results.

IgG Concentration (µg/mL)	Result
0	Negative
0–0.88	Mild allergic reaction
0.88–8.8	Moderate allergic reaction
8.8–88	Highly allergic reaction
88–880	Very highly allergic reaction

**Table 6:** Amino acids and hormones detected in the blood plasma of athletes before and after exercise (FC > 1.5).

No.	Metabolite	Mean Concentration (µM/L), before Loading	Mean Concentration (µM/L), after Recovery	SD before Loading	SD after Loading	Change
1.	2-ketoglutaric acid	1.26	2.68	1.60	4.14	↑2.11
2.	3-Methylhistidine	0.77	0.13	1.01	0.17	↓0.17
3.	Ascorbic acid	1.86	3.19	1.88	3.87	↑1.711
4.	Aspartic acid	2.01	3.16	1.71	2.97	↑1.56
5.	Carnosine	1.26	0.40	0.96	0.18	↓0.31
6.	cis-Aconitic acid	1.29	3.37	1.38	4.38	↑2.59
7.	Citric acid	1.25	4.58	0.09	0.66	↑3.66
8.	Citrulline	2.87	1.48	0.76	0.50	↓0.51
9.	Creatinine	15.14	8.20	-	-	↓0.54
10.	Histidine	34.63	16.82	-	-	↓0.48
11.	Hydroxyproline	2.36	1.10	-	-	↓0.46
12.	Lactic acid	30.04	10.57	3.27	4.14	↓0.35
13.	Lysine	14.97	5.32	-	-	↓0.35
14.	Ornithine	5.07	1.91	-	-	↓0.37
15.	Oxalic acid	5.33	3.13	3.22	1.38	↓0.58
16.	Proline	61.74	33.69	-	-	↓0.54
17.	Serine	25.67	46.73	9.84	14.37	↑1.82
18.	Succinic acid	0.39	0.18	0.30	0.25	↓0.47
19.	Taurine	14.51	28.83	-	-	↑1.98
20.	Threonine	9.64	18.68	-	-	↑1.93
21.	Tyrosine	17.03	6.90	1.60	4.14	↓0.40
	**Hormones**					
22.	11-Deoxycortisol	–	–	–	–	↑1.22
23.	Cortisol	–	–	–	–	↓0.47
24.	Estriol (E3)	–	–	–	–	↑1.43
25.	Dehydroepiandrosterone	–	–	–	–	↓0.43

## Data Availability

Not applicable.

## Supplementary Materials

The following supporting information can be downloaded at: https://www.mdpi.com/article/10.3390/sports11020036/s1, Supplementary S1: Figure S1 “Results of determination of hyper-reactive reaction to food allergens mediated by immune processes”; Supplementary S2: Table S1 “Amino acids detected in the blood plasma of athletes before loading and after loading”; Table S2 “Hormones detected in the blood plasma of athletes before loading and after loading”.
