# Supplementary material for: Molecular Profiling of Athletes Performing High-Intensity Exercises in Extreme Environments
Source: Sports (Basel). 2023 Feb 2;11(2):36. doi: 10.3390/sports11020036 (PMC9963857; doi:10.3390/sports11020036)
Supplement: Supplementary file 1 [file sports-11-00036-s001.zip › Supplementary S1.pdf]

Figure S1. Results of determination of hyperreactive reaction to food allergens mediated by immune processes

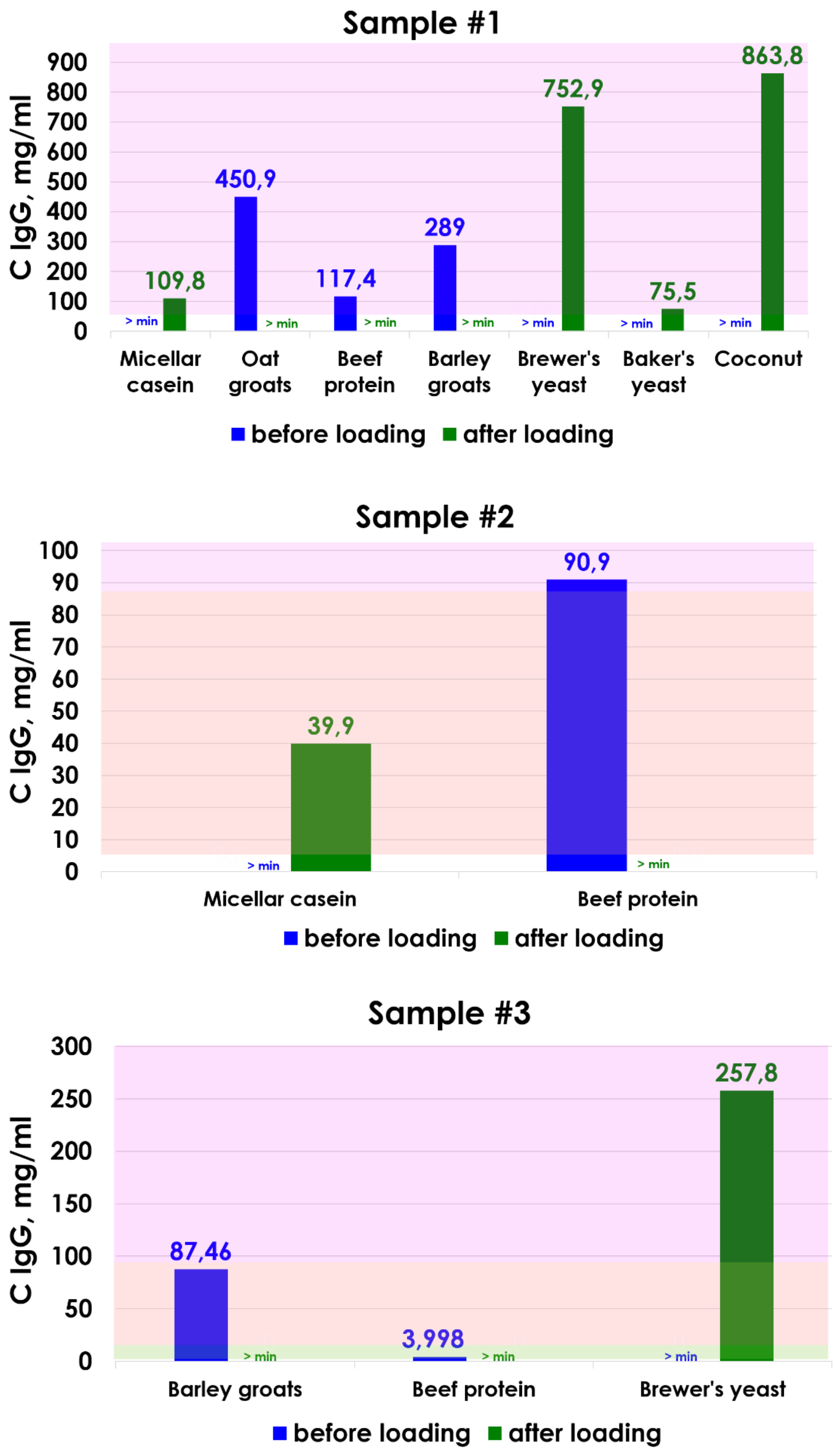

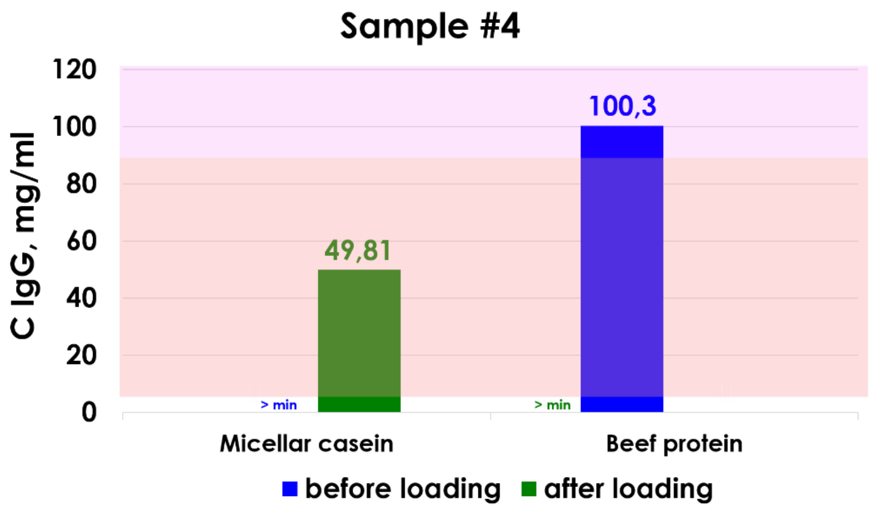
Sample #4
C IgG, mg/ml
120
100
80
60
40
20
0
100,3
49,81
> min
> min
Micellar casein
Beef protein
before loading
after loading

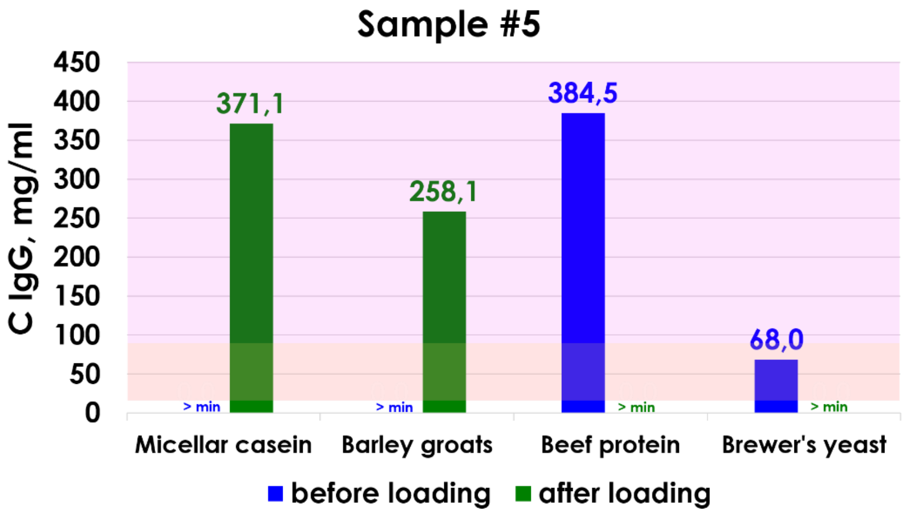
Sample #5
C IgG, mg/ml
450
400
350
300
250
200
150
100
50
0
371,1
384,5
258,1
68,0
> min
> min
> min
> min
Micellar casein
Barley groats
Beef protein
Brewer's yeast
before loading
after loading

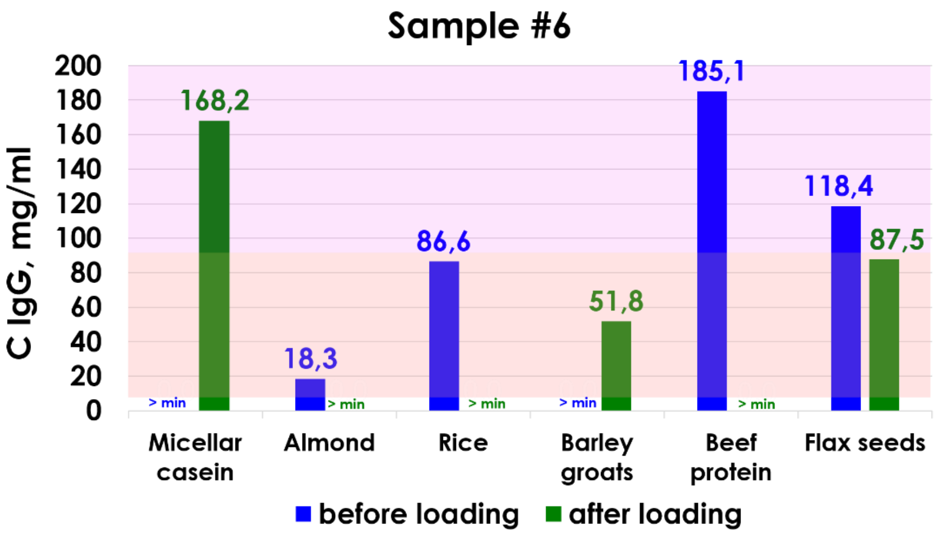
Sample #6
C IgG, mg/ml
200
180
160
140
120
100
80
60
40
20
0
185,1
168,2
118,4
86,6
87,5
51,8
18,3
> min
> min
> min
> min
> min
Micellar casein
Almond
Rice
Barley groats
Beef protein
Flax seeds
before loading
after loading

Sample #7

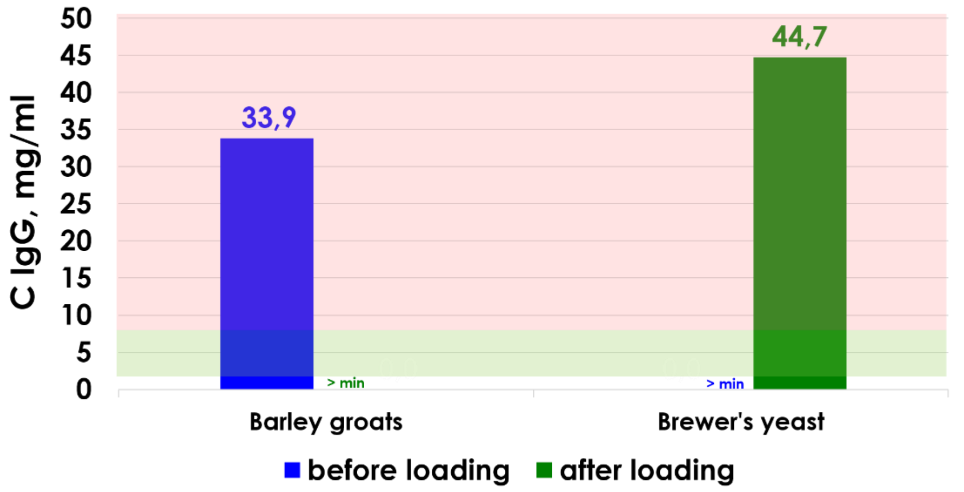

Sample #8

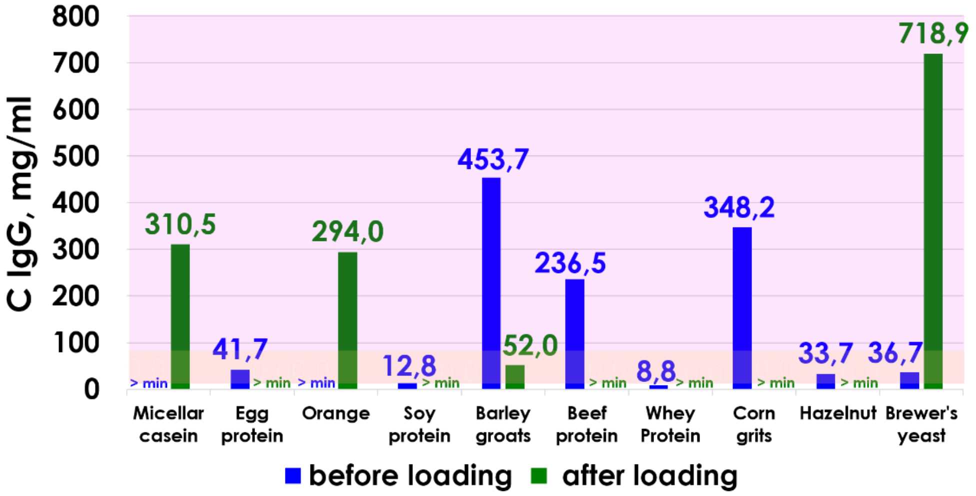

Sample #9

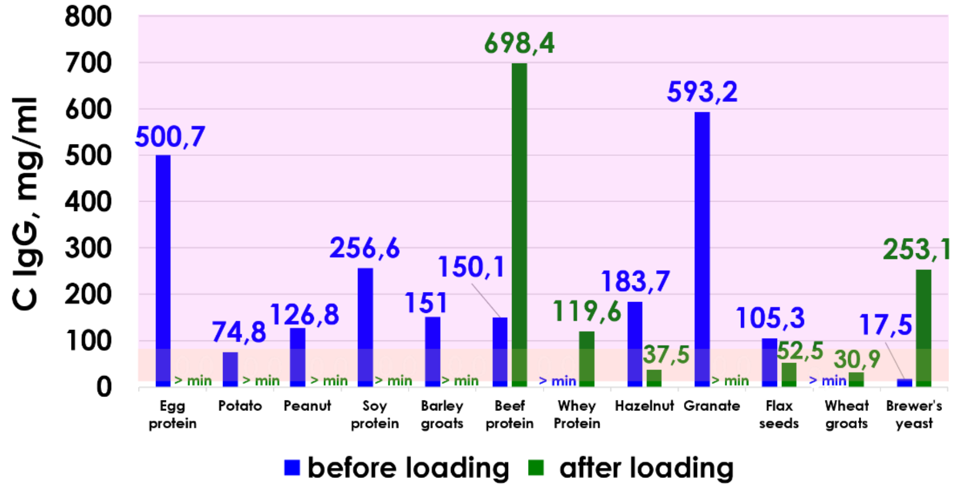

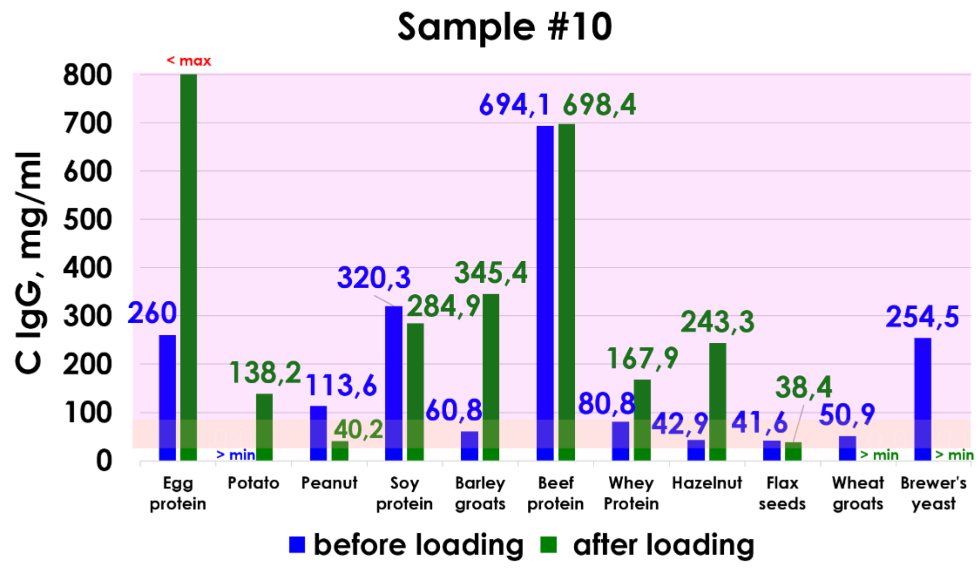
Sample #10
C IgG, mg/ml
800
700
600
500
400
300
200
100
0
< max
260
138,2
113,6
40,2
320,3
284,9
345,4
60,8
694,1
698,4
80,8
167,9
42,9
243,3
41,6
38,4
50,9
254,5
> min
> min
> min
Egg protein
Potato
Peanut
Soy protein
Barley groats
Beef protein
Whey Protein
Hazelnut
Flax seeds
Wheat groats
Brewer's yeast
before loading
after loading

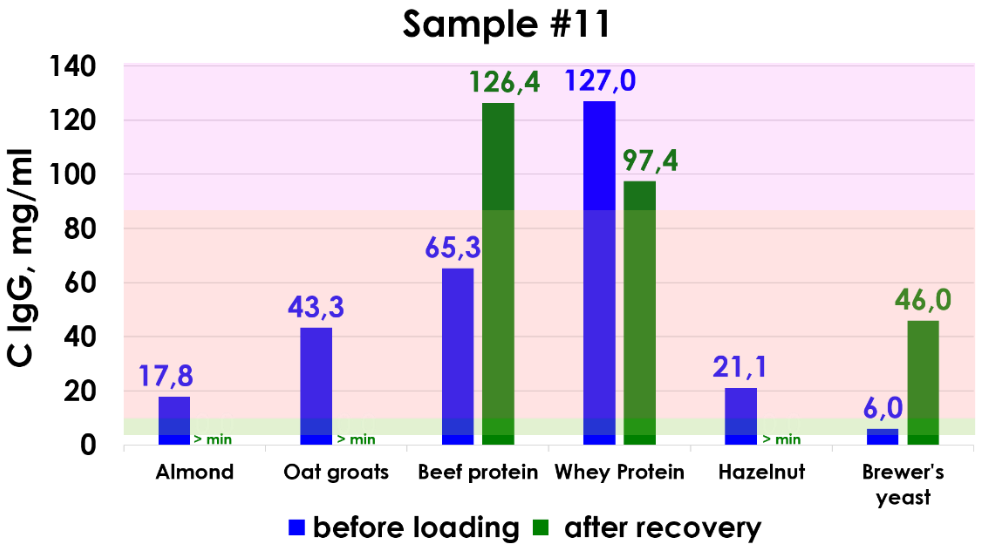
Sample #11
C IgG, mg/ml
140
120
100
80
60
40
20
0
17,8
43,3
65,3
126,4
127,0
97,4
21,1
6,0
46,0
> min
> min
> min
Almond
Oat groats
Beef protein
Whey Protein
Hazelnut
Brewer's yeast
before loading
after recovery
